# Knowledge, Attitude and Practices among Mothers of Children 6 to 24 months of Age Regarding Complementary Feeding

**DOI:** 10.31729/jnma.5274

**Published:** 2020-10-31

**Authors:** Sabina Shrestha, Manoj Pokhrel, Smriti Mathema

**Affiliations:** 1 Department of Pediatrics, Kathmandu Medical College and Teaching Hospital, Kathmandu, Nepal; 2 Department of Obstetrics and Gynaecology, Kathmandu Medical College and Teaching Hospital, Kathmandu, Nepal

**Keywords:** *attitude*, *complementary feeding*, *knowledge*

## Abstract

**Introduction:**

Complementary foods fill the gap between the total nutritional needs of the child and the amounts provided by breast milk. Inappropriate feeding practices are a major cause of the onset of malnutrition in young children. The objective of this study was to assess the knowledge, attitude, and practices of mothers of children between 6 to 24 months of age regarding complementary feeding.

**Methods:**

This Knowledge, Attitude, and Practice Study was conducted among 250 mothers in Kathmandu Medical College and Teaching Hospital from June 2019 to November 2019 after obtaining ethical approval from the institutional review committee (Ref no. 150320199). Convenient sampling method was applied. The mothers of children between 6 to 24 months were interviewed using a structured questionnaire to ascertain the knowledge, attitude, and practices regarding complementary feeding. Statistical analysis was done using SPSS version 20.

**Results:**

Two hundred and fifty mothers were interviewed. 151 (60.4%) mothers knew initiation of breastfeeding soon after birth and 179 (71.6%) were knowledgeable about exclusive breastfeeding for 6 months. 161 (64.4%) mothers knew the proper age of initiating complementary feeding but only 139 (55.6%) mothers practiced it. Early initiation of complementary feeding was done by 87 (34.8%) mothers while 24 (9.6%) mothers delayed it beyond 6 months.

**Conclusions:**

There was a gap in knowledge and practice among mothers regarding adequate age of initiation of complementary feeding, complementary foods, preparation, and practices.

## INTRODUCTION

Complementary feeding (CF) is defined as the systematic process of the introduction of suitable food at the right time in addition to mother's milk to provide needed nutrients to the baby. The World Health Organization (WHO) recommends exclusive breastfeeding for the first six months of life. By 6 months of age, exclusive breastfeeding becomes insufficient to meet the energy and nutrient needs of the growing infant.^1,2^

Children are highly susceptible to growth faltering, especially between 6 and 24 months of age when breast milk is replaced by low nutrient density foods. Moreover, it is difficult to reverse stunting after 2 years.^3^ Therefore, for proper physical growth and neurocognitive development, complementary foods should be introduced timely, adequate in nutrition, appropriate in consistency, in sufficient quantity and hygienic.^4^

The study aims to assess the knowledge, attitude, and practices regarding complementary feeding among mothers with children between 6 months and 24 months of age.

## METHODS

A Knowledge, Attitude, and Practice study was conducted in Kathmandu Medical College and Teaching Hospital over six months from June 2019 to November 2019. Ethical approval was obtained from the institutional review committee of Kathmandu Medical College before initiation of the study (Ref no. 150320199). Mothers having children between 6 to 24 months of age attending the outpatient department of Pediatrics were included in the study. Mothers with children below 6 months or above 24 months and those suffering from chronic illnesses or requiring emergency care were excluded. A convenient sampling method was used and 250 mothers were interviewed during the study period. Data was collected after attaining informed consent from the mothers using a structured questionnaire to ascertain the knowledge, attitude, and practices regarding complementary feeding. Statistical analysis was done using Statistical Package for the Social Sciences (SPSS) version 20.

## RESULTS

Out of the total 250 participants, almost half of the mothers (49.2%) belonged to the age group 26-30 years. The mean age of mothers being 28±4.25 years. Likewise, the maximum number of babies in the study were in the age group 12-18 months with the mean age being 13.95 ± 5.84 months. There were 127 (50.8%) male babies and 123 (49.2%) female babies. Nuclear family system 161 (64.4%) was more common than the joint family 89 (35.6%). Most of them were from urban areas 219 (87.6%) followed by semi-urban areas 19 (7.6%) and rural areas 12 (4.8%). The sociodemographic profile of the participants is shown (Table 1).

**Table 1:** Socio-demographic profile of the participants.

Characteristics	Frequency n (%)
**Age of the child (in months)**	
6-8	55 (22)
9-11	31 (12.4)
12-18	109 (43.6)
19-24	55 (22)
**Sex of the child**	
Male	127 (50.8)
Female	123 (49.2)
**Age of mother (years)**	
≤ 20	9 (3.6)
21-25	59 (23.6)
26-30	123 (49.2)
31-35	44 (17.6)
>35	15 (6)
**Mother’s educational status**	
Illiterate	17 (6.8)
Primary	41 (16.4)
Secondary	75 (30)
Intermediate	39 (15.6)
Graduation	78 (31.2)
**Mother’s occupational status**	
Housemaker	154 (61.6)
Service	59 (23.6)
Business	37 (14.8)
**Type of family**	
Nuclear	161 (64.4)
Joint	89 (35.6)
**Child size per family**	
1-2	195 (78)
>2-4	55 (22)
**Residence**	
Rural	12 (4.8)
Urban	219 (87.6)
Semi-urban	19 (7.6)

On assessing the mother's knowledge on infant and young child feeding, 151 (60.4%) mothers knew that breastfeeding should be initiated soon after birth, while 9 (3.6%) had no idea about it. Most of the mothers 179 (71.6%) were knowledgeable regarding exclusive breastfeeding upto 6 months of age. 161 (64.4%) mothers knew about the proper age of initiation of complementary feeding and 172 (68.8%) knew that complementary food should be given thrice a day. Likewise, most of the mothers 178 (71.2%) knew home-made complementary food while 34 (13.6%) knew about commercially available complementary food and 38 (15.2%) knew about both home-made and commercially available complementary food. However, only 120 (48%) mothers had knowledge about iron-rich food while 178 (71.2%) of them knew about adding iodized salt to the complementary food. The mother's knowledge of infant and young child feeding is shown (Table 2).

**Table 2:** Mother’s knowledge of infant and young child feeding.

Characteristics	Frequency n (%)
**Initiation of breastfeeding**	
Soon after birth	151 (60.4)
1 day after birth	69 (27.6)
2-3 days after birth	21 (8.4)
No idea	9 (3.6)
**Exclusive breastfeeding**	
Upto 6 months	179 (71.6)
4-5 months	46 (18.4)
2-3 months	13 (5.2)
Upto 1 month	9 (3.6)
No idea	3 (1.2)
**Initiation of complementary feeding**	
4-5 months	71 (28.4)
At 6 months	161 (64.4)
At 8 months	18 (7.2)
**Frequency of complementary feeding**	
Twice a day	78 (31.2)
Thrice a day	172 (68.8)
**Knowledge about complementary feeding**	
Homemade	178 (71.2)
Commercially available	34 (13.6)
Both	38 (15.2)
**Source of knowledge about complementary food**	
Health professional	53 (21.2)
Family	172 (68.8)
Electronic media	6 (2.4)
Relatives	19 (7.6)
**Knowledge about iron-rich food**	
Yes	120 (48)
No	130 (52)
**Knowledge about adding iodized salt**	
Yes	178 (71.2)
No	72 (28.8)

Most of the mothers 226 (90.4%) think that dietary diversity should be considered while feeding infants and young children. But regarding feeding during episodes of illness, most of the mothers 134 (53.6%) think that the quantity and frequency of food should be decreased, 27 (10.8%) think that the quantity and frequency should be withheld, 65 (26%) mothers believe that the same quantity and frequency of food should be maintained even during illness, while 24 (9.6%) mothers think that it should be increased instead. Regarding cultural and social food taboos, 147 (58.8%) mothers consider banana, yogurt and rice as cold food, 42 (16.8%) mothers believe that meat, pulses, nuts, and eggs are hot and hard to digest while 45 (18%) believe in both of these food taboos. There were only 16 (6.4%) mothers who did not believe in any social and cultural food taboos. Maximum mothers 207 (82.8%) preferred homemade foods as complementary food and also preferred preparing separate complementary food for children. The attitude of mothers regarding infant and young child feeding is shown (Table 3).

**Table 3:** Mother’s attitude on infant and young child feeding.

Characteristics	Frequency n (%)
**Dietary diversity**	
Yes	226(90.4)
No idea	24 (9.6)
**Complementary feeding practices and frequency during illnesses**	
Decrease quantity and frequency of food	134 (53.6)
Withhold quantity and frequency of food	27 (10.8)
Maintain the same quantity and frequency of food	65 (26)
Increase the quantity and frequency of food	24 (9.6)
**Cultural and social food taboos**	
Banana, yogurt, and rice as cold food	147 (58.8)
Meat, pulses, nuts, and eggs are hot and hard to digest	42 (16.8)
Both	45 (18)
Does not believe in food taboos	16 (6.4)
**Preferences about complementary food**	
Homemade	207 (82.8)
Commercially available foods	19 (7.6)
Both	24 (9.6)
**Preferences about the preparation of complementary food**	
Prepare separate complementary food for children	207 (82.8)
Prepare combinedly as adult food	43 (17.2)

Almost all the mothers who participated in the study practiced hand washing before preparing food and used boiled water for drinking. Timely initiation of complementary food at 6 months of age was practiced by 139 (55.6%) mothers only. 87 (34.8%) mothers introduced complementary food earlier than 6 months while 24 (9.6%) started complementary feeding after 6 months of age. The reason for the delayed introduction of complementary food was because they thought milk was enough, the child vomited everything when started on complimentary food, an elder in the family told to do so and few said that the child did not accept the food. Most of the mothers 210 (84%) fed their children three times a day while 40 (16%) of them fed only twice a day. Likewise, thick consistency complementary food was fed by 137 (54.8%) mothers only while 113 (45.2%) mothers prepared thin gruels. The mother's practices regarding complementary feeding are shown (Table 4).

**Table 4:** Mother’s practices on complementary feeding.

Characteristics	Frequency n (%)
**Washing hands before cooking**	
Yes	250 (100)
No	0
**Boils drinking water**	
Yes	240 (96)
No	10 (4)
**Initiation of complementary feeding**	
Before 6 months	87 (34.8)
At 6 months	139 (55.6)
After 6 months	24 (9.6)
**Reason for delayed complementary feeding**	
Vomits everything	2 (0.8)
Milk is enough	17 (6.8)
Elder told to do so	2 (0.8)
The child did not accept other food	3 (1.2)
**Types of complementary food**	
Commercial food as complementary food	25 (10)
Litto	27 (10.8)
Jaulo	36 (14.4)
Rice, dal, vegetables	20 (8)
Commercial food and litto	21 (8.4)
Commercial food and jaulo	6 (2.4)
Litto and jaulo	34 (13.6)
Commercial food, litto, jaulo	15 (6)
Jaulo and rice, dal, vegetables	16 (6.4)
All (litto, jaulo, rice)	50 (20)
**Frequency of complementary feeding**	
Thrice a day	210 (84)
Twice a day	40 (16)
**Consistency of complementary food**	
Thick	137 (54.8)
Thin	113 (45.2)

## DISCUSSION

The period of complementary feeding between 6 and 24 months of age is considered to be a crucial window of opportunity for preventing undernutrition and its long term negative outcomes in infants.^5^ According to the WHO guidelines, complementary feeding should be started at 6 months of age, while continuing breastfeeding up to 2 years or more.^6,7^ Introduction of complementary foods at an appropriate age is essential for proper physical growth and neurocognitive development. Initiating complementary feeding too early may increase the risk of gastrointestinal and respiratory tract infections, weight gain during infancy, and obesity in later life. The late introduction of complementary feeding may increase the risk of nutritional insufficiency, immune disorders, and type 1 or 2 diabetes in later life among high-risk populations.^8^

In this study, we tried to establish the duration of breastfeeding, the age at which complementary feeding was started, what common foods were given as complimentary food, and various other aspects related to complementary feeding.

The World Health Organization (WHO) recommends exclusive breastfeeding for the first six months of life.^2^ This study showed that the knowledge about exclusive breastfeeding was high (71.6%) and initiation of breastfeeding soon after birth was satisfactory (60.4%). Likewise, 64.4% of mothers knew about adequate time for initiation of complementary feeding but only 55.6% were found to be practicing it. This shows that there is a gap between knowledge and practice about infant and young child feeding. Even a higher gap in knowledge and practice regarding initiation of complementary feeding was found in a study conducted in Kanti Children's Hospital in Nepal in 2011.^9^

In our study, early initiation of complementary feeding was found in 34.8%. One of the major reasons for early initiation may correlate to the cultural practice among Nepalese people of introducing semi-solid food in a ceremony known as rice feeding ceremony or Pasni which is usually done at 4-5 months of age.^10^ Once introduced, people tend to continue with semi-solid foods thereafter. Similarly, early initiation was still high in another study conducted in Dhaka, Bangladesh.^11^

It was also found that 9.6% of mothers delayed initiation of complementary feeding beyond 6 months of age. The most common reasons given by them were enough milk production, non-acceptance of other foods by the baby, vomiting when other foods were given, and some delayed complementary feeding as per the advice given by the elders in the family. A study done in tertiary care hospital of Nepal showed similar reasons for the delayed introduction of complementary food.^9^

In one study conducted in Nepal in 2015, 77.7% of mothers knew that complementary feeding should be started at 6 months of age. However, irrespective of this knowledge, only 50% of the mothers started complementary feeding at 6 months of age. In the same study, 40.3% of the mothers started complementary feeding before the recommended age and 9.7% delayed it beyond 6 months of age.^12^

The mother's preferences on the type of complementary food were varied. However, the study showed that they preferred homemade food preparations as compared to commercially available complementary food. This may be because family members were the major source of knowledge regarding complementary foods. The other sources of knowledge about complementary foods were health professionals, relatives, and electronic media. However, the use of commercially available complementary foods is also increasing which may be because of easy availability, easy preparation, and aggressive marketing policies.

The study also found that 210 (84%) mothers were giving complementary food thrice a day, but a thin consistency complementary food was prepared by 113 (45.2%) mothers. In a study done in India, 25.5% to 30% of mothers knew and practiced the proper consistency of complementary feeding, which is still lower than the finding in our study (54.8%).^13,14^ Though the frequency of feeding is adequate, the food preparation may be deficient in calories and nutrients as most of the mothers were preparing thin consistent complementary foods. This might be one of the factors responsible for the high prevalence of undernutrition, wasting, and stunting in our country.

This was a hospital-based study and the result might not reflect the values present in the general population, for which population-based or community-based studies might be necessary. Information was taken from mothers on a recall basis, which may have resulted in bias, as the recall information may not be accurate.

## CONCLUSIONS

The knowledge regarding timely initiation of complementary feeding among the mothers is inadequate and feeding practices are inappropriate. The initiation of CF is ideal in only half of the respondents with majority initiating complementary feeding before 6 months of age. False beliefs as well as social and cultural taboos tend to wean the child at an inappropriate age and prevent consumption of nutritious food.

Hence, it is essential to provide proper knowledge and education to mothers and caregivers regarding appropriate timing of initiating complementary feedings, complementary foods, preparation, and practices to prevent malnutrition and improve the health status of children.

## Conflict of Interest

**None.**
